# Person-centered care model in dentistry

**DOI:** 10.1186/s12903-018-0661-9

**Published:** 2018-11-29

**Authors:** Hyewon Lee, Natalia I. Chalmers, Avery Brow, Sean Boynes, Michael Monopoli, Mark Doherty, Olivia Croom, Lilly Engineer

**Affiliations:** 1 DentaQuest Institute, 10320 Little Patuxent Pkwy., Suite 200, Columbia, MD 21044 USA; 2 DentaQuest Institute, 2400 Computer Dr, Westborough, MA 01581 USA; 3 DentaQuest Foundation, 465 Medford St, Boston, MA 02129 USA; 4 Safety Net Solutions, DentaQuest Institute, 2400 Computer Dr, Westborough, MA 01581 USA; 5 Department of Anesthesia and Critical Care Medicine, Johns Hopkins School of Medicine, Baltimore, MD 21205 USA; 6 Department of Health Policy and Management, Johns Hopkins Bloomberg School of Public Health, 600 N. Wolfe St, Baltimore, MD 21205 USA; 7 Present address: U.S. Food and Drug Administration, Silver Spring, USA; 8 AcademyHealth, 1666 K Street NW, Suite 1100, Washington, DC, 20006 USA

**Keywords:** Oral health, Person-centered care, Dentistry, Integrated care

## Abstract

**Background:**

To achieve optimal health and oral health, the system of care must place a person and their social well-being at the center of decision making and understand factors spent outside the clinical settings, including individual behavior, context and lifestyle.

**Main text:**

Person-centered care offers a unique and compelling opportunity for dentistry, and its practitioners, to improve quality of care and overall health outcomes. For decades, the dominant treatment modalities within dentistry primarily focused on a surgical, treatment-oriented approach as opposed to health promotion and improvement. However, new business and care models are disrupting the dental care system, and transforming it into one that is focused on disease management and prevention-oriented primary care that considers overall health and well-being. We proposed a person-centered care model to improve oral health as an integral part of overall health. The model identified three key players who act as change agents with their respective roles and responsibilities: Person, provider, and health care system designer.

**Conclusions:**

While previous person-centered models in dentistry focused on the role of providers within the clinical setting, this work emphasizes the role of the care designer in creating an environment where both person and provider are able to communicate effectively and achieve improved health outcomes.

## Background

### Oral health disparity: a silent epidemic in the United States

**Fig. 1:**
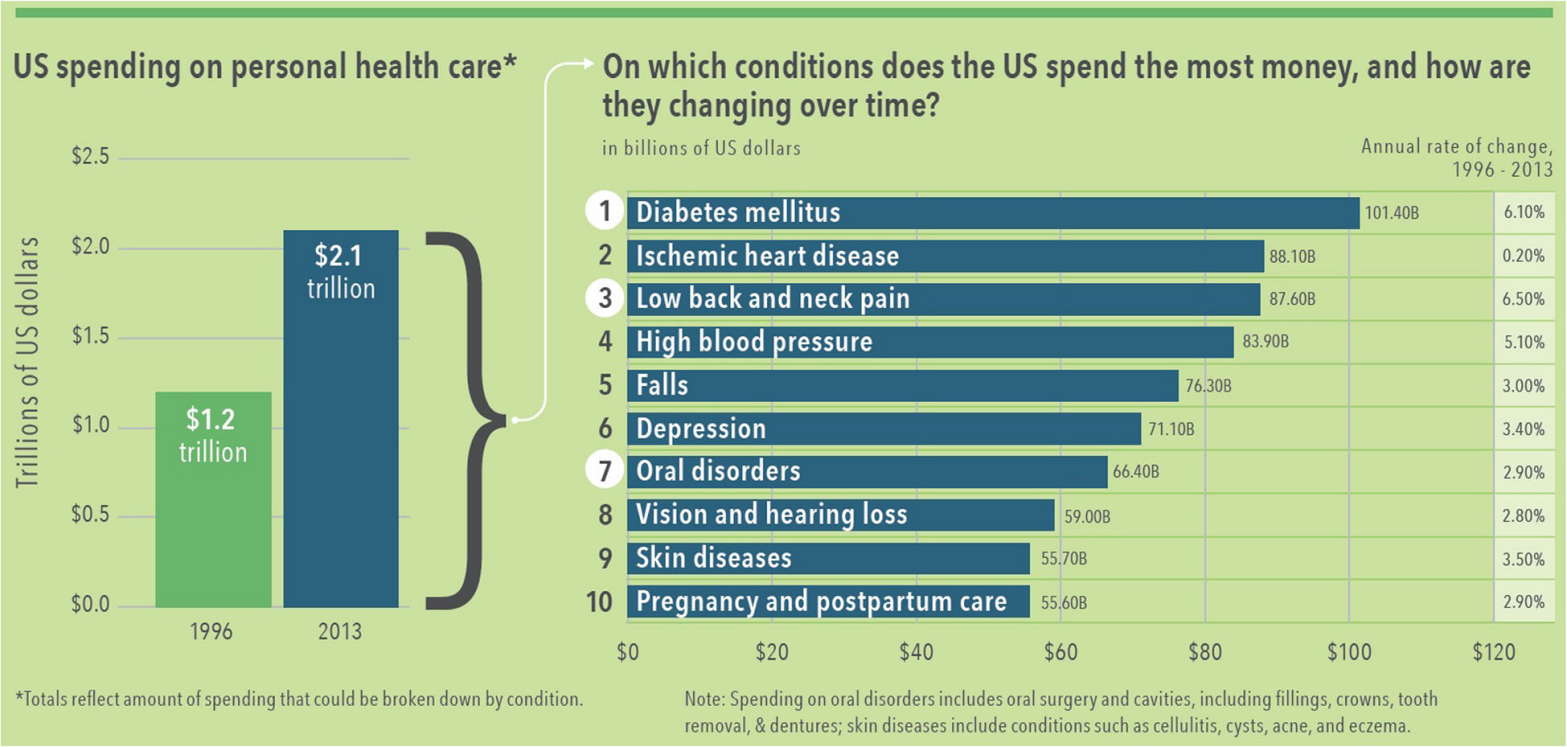
US Spending on Personal Health Care and Public Health, 1996–2013 [11]

Dental caries, or tooth decay, is a transmissible yet preventable chronic disease that is prevalent worldwide and afflicts individuals of all ages [1–3]. Despite increased attention by health organizations and health care systems, caries remains mostly a silent epidemic with a significant impact on the nation’s general health and well-being. Periodontitis, a result of inflammation in the gums and bone tissue that surround and support the teeth, is a second common chronic disease of the oral cavity. Almost half of adults, aged 30 and older, have some form of periodontal disease, while older people and those with a lower socio-economic status are disproportionately affected [4]. In the United States alone, more than $113 billion is spent on dental care annually [5]; more than an estimated $6 billion in productivity costs are lost each year due to employee absenteeism related to dental issues [5]. Consequences of poor oral health can also negatively influence speech, nutrition, growth and function, and social development, and is associated with difficulty in obtaining employment and underperformance in academic and employment settings [6–9]. National health expenditures are projected to continue to grow exponentially and will represent 19.9% of the United States gross domestic product by 2025 [10]. Oral disorders remain in the top ten most expensive conditions, when accounting for personal health care spending nationally (Fig. 1) [10–12].

### Person-centered care: from treating diseases to promoting health

To achieve optimal oral health, the system of care must place a person and their social well-being at the center of decision making, including the understanding of factors spent outside the dental office [13]. Medical care, genetics, and individual biology account for less than one-third of all determinants of health; this means that better overall health lies in addressing additional factors including individual behavior, environmental and social circumstances of patients [14, 15]. Therefore, context and lifestyle have significant roles in improving and maintaining optimal oral health.

To improve health outcomes for patients, the National Academy of Medicine (NAM; formerly the Institutes of Medicine) recognized the need for a patient-centered model of health care. The NAM thus defined the concept of patient-centered care as the provision of care “that is respectful of and responsive to individual patient preferences, needs, and values, and ensuring that patient values guide all clinical decisions” [16]. More recently, NAM focused on the integration of oral health and primary care as well as particularly challenging aspects of bridging oral health and primary care for populations with low health literacy [17]. The implementation of patient-centered care has subsequently demonstrated reductions in annual overall medical care charges [18], the promotion of effective change for patients and health care professionals, and improvements in patient satisfaction [19, 20]. This model of care has also shown improvements in overall patient health when they transition from pediatric to adult primary care [21].

A related but distinct approach, person-centered care has since developed, marking a transition from the medical patient, to the whole person. This shift in focusing on the person rather than the patient is based on the “accumulated knowledge of people, which provides the basis for better recognition of health problems and needs over time,” and helps to “facilitate appropriate care for these needs in the context of other needs” [22]. Person-centered care developed out of the nursing, gerontology and long-term care fields [23] as each recognized the growing importance of a person’s living environment, resources and self-management capacity in predicting disease outcomes. This approach has resulted in improvements in addressing chronic conditions and increased patient satisfaction with care providers, as well as concurrent reductions in the likelihood of treatment failures [13].

## Main text

### Oral health as an integral part of overall health

Person-centered care offers a unique and compelling opportunity for dentistry and its practitioners to improve the quality of care and overall health outcomes. For decades, the dominant treatment modalities in dentistry primarily focused on a surgical, treatment-oriented approach as opposed to health promotion and improvement. However, new business and care models are disrupting the dental care system, and transforming into one that is focused on disease management and prevention-oriented primary care that considers overall health and well-being.

Mounting evidence shows the bi-directional relationship between oral health and other systemic diseases like diabetes [24–29]. Studies have shown that patients with diabetes have increased prevalence, severity and accelerated progression of periodontitis compared to those without diabetes [25, 27, 28]. Also, uncontrolled periodontitis negatively affects glycemic control in patients with diabetes [26, 29], and periodontal intervention may reduce medical costs and improve health outcomes among individuals diagnosed with type 2 diabetes [30, 31]. Designing and implementing a person-centered care model in dentistry, dental professionals are supported by a system of care that reinforces collaboration with other health care professionals in improving the overall health and well-being of person with diabetes. Oral diseases also share common causes with other non-communicable and chronic diseases and risk factors, like high sugar intake and smoking.

Despite the importance of oral health as an integral part of overall health, oral health is frequently omitted from disease management plans and health education due to the historic separation between medicine and dentistry. Multiple person-centered care models are used globally but they lack consensus concerning a basic definition of person-centered care within dentistry due to varied interpretations and applications of the concept [32, 33]. Some suggest a conceptual-based approach while others propose a clinically-based model for person-centered care. Person-centered care and patient-centered care, though separate concepts, are used interchangeably without a clear distinction between the two [32–34]. Moreover, there is limited evidence demonstrating improved oral health outcomes with a person-centered approach in dentistry, compared to medicine [33, 34].

To prevent any health risks originating from poor oral health and to improve overall health and well-being, a person-centered care approach that integrates oral health into overall health must be a critical element in both care design and delivery.

### Proposed person-centered care model in dentistry: Person, provider, care designer

Person-centered care starts with learning the contextual elements surrounding and shaping a person’s behavior, decisions, and barriers to health. Then, the person-provider team applies that contextual knowledge to develop opportunities that will help attain the best health outcomes possible. Health care system designers should empower the person-provider team through the development and function of health care systems where this team can achieve improved health outcomes. Care designers are entities and systems rather than personnel who create infrastructure for the person-provider team. Examples include hospitals and clinics, community organizations, public or private medical and dental insurance entities, and the local, state and federal government. Their primary role is to design and operate a system of care that contextually assures the person-provider relationship forms in the most meaningful and efficient way. This model identifies three key players as change agents and the respective roles and responsibilities (Table 1) [35].

**Table 1:** Person-Centered Care Model: Three Key Players and Their Roles and Functions

ACTION	Person or Primary Caretaker	Provider – Coach	Care Designer
Lean Examine	Learn about his or her oral health status during the initial exam and periodic follow-up, which could take place outside a dental office or by a non-dentist member of the treatment team	Learn about the person’s oral health and medical health as supported by examination and medical records	Learn and examine current person-centered care models in both dental and non-dental healthcare fields
Relate Share	Relate their oral health and general health	Relate oral health with other medical conditions Share findings and interconnections between oral health and overall health with the person and other healthcare providers as necessary	Relate those successful approaches to revise and expand its operations and services to achieve its mission Share opportunities and new findings internally
Plan Design	Plan for preventive intervention, definitive treatment, behavior modification and lifestyle changes with providers	Plan for preventive interventions, definitive treatments, and behavior modifications that the person agrees upon	Design or redesign a system of care to improve health outcomes, reduce cost of care, and increase satisfaction of the care experience for both patient and providers [35] Design financial incentives and alternative payment designs that reflect evidence-based dental practice and knowledge.
Act Provide Track Evaluate	Act upon the agreed plan	Provide preventive interventions, definitive treatments, and behavior modifications that the person agrees upon, using available tools, techniques and clinical support; Track and Evaluate the progress of the person’s adherence to the agreed plan	Implement a designed system of care. Track the progress of implemented system designs Evaluate progress and outcomes
Revise	Revise the plan and adjust as necessary to achieve meaningful yet practical goals in consultation with his/her provider.	Revise the plan together with the person to achieve the set goals.	Revise the design of the care system by incorporating the voices of persons and providers, as well as analyzes, shares and applies any emerging knowledge gleaned.

Within a person-centered care environment, a person is a recipient of care, and can act as a partner who co-designs his/her care delivery. Additionally, families and caregivers are often involved in the course of treatment and present during interactions with providers, and they can actively engage in care policy and practice improvement through patient and family advisory councils. Providers include health care professionals, community outreach personnel, clinical and administrative staff, who interact directly or indirectly with the person to achieve a common goal: improving oral health and the overall health of the person. Depending on the nature of disease and health concerns, a multidisciplinary team of health professionals can interact with the person in both clinical and non-clinical settings. To empower this person-provider team, care designers must develop value-supported systems, encourage personal health ownership, and create financial structures and payment environments that incentivize health. Sophisticated payment design may include co-incentives for medical and dental teams when a person’s health outcomes improve through application of person-centered medical and dental care.

The evaluation of a person-centered care system’s success can be aligned with major health quality measures already in use. These include measures endorsed by the National Quality Forum (NQF) outlined in Healthy People 2020, as well as other measures based on patient satisfaction surveys and quality of life assessments and evaluations. Additionally, care designers must also demonstrate that their person-centered care models directly improve population health and develop specific goals for improvement, especially for persons with chronic conditions. This framework is applied in Table 2 showing the utilization of this approach for a person with diabetes.

**Table 2:** Person-Centered Care in Dentistry: Person-Centered Care for Person with Diabetes

Person	Providers	Care designer
Learn • Learn current oral health status and problems • Learn optimal oral health hygiene habits, and diet pattern	Learn/Examine • Learn about the person’s oral health and diabetes status supported by medical records and a clinical examination • Learn personal barriers making it difficult to achieve optimal oral health and diabetes control • Perform diabetes screening and check blood glucose level or HA1c	Learn/Examine • Learn and examine existing oral health care models and person-centered care models for people with diabetes • Learn the geographical context (state law, coverage, oral health and health needs) to design a care model that is the best fit for the context
Relate • Understand how oral health is related to diabetes • Relate daily oral hygiene habits, and diet pattern to oral health	Relate • Relate oral health conditions to current diabetes status and other medical conditions • Relate a person’s barriers to ideal care to a future plan Share • Communicate with the person about the oral-systemic link in context of diabetes • Share both oral and medical findings with a person’s primary care provider team as necessary and inform them about the oral-systemic link in diabetes context. • Share how the person can improve oral health and diabetes status both in and out of clinical settings.	Relate • Relate those successful person- centered care models in designing operative system • Relate current models to the target community and state demographic and characteristics Share • Share opportunities to implement person-centered care in people with diabetes with internal stakeholders • Develop an oral-systemic link message for providers to share with persons with diabetes • Share person-centered care model outcomes with both internal and external partners
Plan • Set personal oral health goals and plan out actionable items • Decide on treatment plan with provider	Plan • Present treatment and behavior modification plan to the person for an informed decision	Design • Design a system that incentivizes person-provider teams who meet key person-centered care measures • Design a coordinated care system that exists between medical and dental providers
Act • Actively participate in the agreed plan in both prevention and treatment procedures • Modify oral hygiene habits and diet pattern as planned outside of the clinical setting	Provide • Provide preventive and or definitive care as planned Track • Track the person’s adherence to the agreed plan, progress on oral health improvement and diabetes control Evaluate • Evaluate the progress on pre-determined evaluation measures	Implement • Implement demonstration projects to improve health of the diabetic population, a reduction in both medical and dental costs, and an increase in satisfaction of the care experience of both person and providers Track • Track the progress of the implemented person-centered care model • Support person-provider team to track the progress using health information technology and personalized health platform Evaluate • Develop a set of evaluation tools that are aligned with major oral health and diabetes measures
Revise • Revise plans based on personal experience, diabetes status, and oral health outcomes from the current plan	Revise • Revise the plan with the person to achieve the set goals or to modify the goal	Revise • Revise the person-centered care model incorporating reflections from people with diabetes and providers • Modify person-centered care models for diabetes to improve care outcomes, costs, and care experience

Source: The person-centered care approach to improving the oral health of all: A Framework for DentaQuest

Other chronic conditions may also benefit from the application of this approach. Dental caries is an infectious disease and cariogenic bacteria can be transmitted from caregivers to young children; this early transmission increases children’s risk for the disease [36]. Translating this scientific knowledge to practice, care designers are able to create systems of care that provide oral health education and treatment to pregnant women so they can modify their lifestyle and receive dental services prior to giving birth. Providers can address specific concerns related to pregnant women through this person-centered care approach, such as the infectious nature of oral disease, morning sickness, prevention of erosion and others. Providers can also consider treatment plan modifications and possible use of alternative medicines that are based on the unique physiology of pregnant women.

Person-centered care is not limited to the structural boundaries of the clinics. For children who reside in communities and with difficulty accessing oral health care, care designers can collaborate to offer alternative treatment modalities in non-traditional clinic settings, like school-based clinics or mobile dental services. These can be feasible options for individuals with a lack of structural and geographical access to dental services. With all three participants in this person-centered approach, patient health and safety are optimized and risks are minimized.

### Optimization of a person’s health: an integrated systems approach

Treatment and management of oral disease often requires coordination of care beyond the delivery of preventive and restorative treatments at the dental facility. Transportation, navigating care, and collaboration with other multidisciplinary team members to address both oral and overall health are also necessary [37]. Vital to success is ensuring a system design that respects the dignity of the person and allowing for structured access to desired and required health care.

Previous person-centered care models within dentistry owed lack of success in part to only a defined role of providers in clinical settings. Our model highlights the essential role of the care designer within the broader system to create environments and vehicles for providers to practice a person-centered approach in the most meaningful and effective ways. The person-centered approach in dentistry must include the care designer as an active and competent player for a sustained system benefit. Without support systems, coverage, or incentives, neither the person nor provider can pursue a person-centered care approach.

### Future challenges

Existing challenges to person-centered care within dentistry are substantial and include limitations in health information technology, particularly a lack of medical-dental electronic record interoperability, and a lack of effective models for care coordination. There are insufficient sociodemographic information collection mechanisms in dentistry from either individuals or provider. By investing in, and strengthening existing health information technology platforms, and beginning the integration of predictive data analysis using consumer-based, sociodemographic data, care designers and providers can begin to overcome some of these inherent challenges that currently hinder widespread adoption of person-centered care models.

Designers should identify key stakeholders and partners who understand the person-centered care, the existing challenges encountered by patients, and who share a common vision to demonstrate the value found in utilizing this approach. Focused person-centered care demonstration projects can show improvement in health outcomes and care experiences in specific contexts. Thus, care designer’s person-centered care models aim for adaptability, which means that inputs from the person-provider team can, and should, modify the model periodically and when needed.

### Position oral health as primary care

The crucial final strategy for this person-centered approach in dentistry is to position oral health as an integral and necessary part of primary health care services and attainment. In recent years, multiple health care organizations and clinical leaders have recognized the important role oral health has in primary health care and highlight the integral part it plays in overall health [38–41]. In addition, recommendations have been established by the Health Resources and Services Administration (HRSA) that set forth competencies for inter-professional oral health practices [42]. Primary care teams have the experience, skills and close relationships with community members that allow them to accomplish effective prevention and disease management. A paradigm shift in the common perception of oral health care from a surgical treatment-oriented approach to one that is focused on disease management and prevention-oriented primary care is proposed, and this shift is reflected in both treatment and the existing financial models.

## Conclusions

The impact of disparities in oral health and the historical chasm between medicine and dentistry are important considerations in realizing the person-centered care approach in dentistry. While previous person-centered models in dentistry focused on the role of providers within the clinical setting [32–34], this work identifies three key players: person, provider and the care designer. To understand the contextual circumstances of individuals as well as that of practice management, the role of the care designer as a system enabler is essential, especially in creating an environment where both person and provider are able to communicate effectively. Aligning evaluation of person-centered care within other major national health measures improvements in health outcomes and cost-effectiveness to promote overall health and well-being of “a person” as well as a “population” can be realized.

## Abbreviations

HRSA: Health Resources and Services Administration
NAM: National Academy of Medicine (formerly the Institutes of Medicine)
NQF: National Quality Forum
